# RNA-binding protein HnRNPU regulates proliferation and ferroptosis in colon adenocarcinoma by stabilizing the mRNA of system xc^−^

**DOI:** 10.1038/s12276-025-01569-z

**Published:** 2025-11-28

**Authors:** Yu Zhang, Qingkun Wang, Yue Han, Yubing Wang, Lei Zhao, Houkun Zhou, Ruixue Zhang, Shuhao Wang, Xiuying Jin, Junjie Piao

**Affiliations:** 1 Key Laboratory of Pathobiology, Yanbian University, State Ethnic Affairs Commission, Yanji, China; 2 Central Laboratory, The Affiliated Hospital of Yanbian University, Yanji, China

**Keywords:** Prognostic markers, Colon cancer

## Abstract

Ferroptosis is a distinct form of programmed cell death that differs from other pathways. It is characterized by iron-dependent lipid peroxidation and results in morphologically lethal cellular damage. With advancing insight, triggering ferroptosis is a promising strategy for cancer therapy. RNA-binding proteins (RBPs) comprise a diverse group of molecules that regulate various RNA processes through interactions with transcripts. Research has highlighted the pivotal role of RBPs in controlling biological functions. Evidence indicates that RBPs play important roles in regulating ferroptosis. Heterogeneous nuclear ribonucleoprotein U (HnRNPU) is a well-known RBP involved in RNA splicing, messenger RNA stability and chromatin organization. Elevated HnRNPU expression has been implicated in cancer progression and is associated with poor prognosis. However, the function and underlying mechanisms of HnRNPU in colon adenocarcinoma (COAD) remain poorly understood. Here we identify increased HnRNPU expression in patients with COAD, with higher levels correlating with poor patient survival. HnRNPU knockdown inhibited cell proliferation and induced cell cycle arrest by suppressing cyclin E1 and CDK2. RNA-sequencing analysis revealed HnRNPU’s involvement in ferroptosis regulation. In line with this, HnRNPU deletion induced ferroptosis and increased sensitivity to RSL3 treatment and cysteine deprivation. xCT overexpression (SLC3A2/SLC7A11) counteracted the antiproliferative and proferroptotic effects of HnRNPU knockdown. Mechanistically, HnRNPU stabilized the mRNAs of SLC7A11 and SLC3A2 by binding to their 3′ untranslated regions, thereby promoting cysteine uptake and glutathione synthesis. Findings demonstrate that HnRNPU promotes proliferation and inhibits ferroptosis by regulating the mRNA stability of SLC7A11 and SLC3A2. Targeting HnRNPU is a potential therapeutic approach for COAD treatment.

## HnRNPU drives cancer growth by inhibiting ferroptosis

Ferroptosis is a type of cell death caused by too much iron in cells and linked to diseases such as cancer. This study looks at a protein called HnRNPU in colon cancer. They found that HnRNPU is more common in cancer tissues and is linked to worse outcomes for patients. The researchers used various lab techniques to study colon cancer cells. They reduced HnRNPU levels in these cells and observed that it slowed cell growth and triggered ferroptosis. This happened because HnRNPU affects the stability of certain messenger RNAs, which are crucial for cell survival. By destabilizing these mRNAs, the cells could not take up cystine, a nutrient needed to prevent ferroptosis. Study suggests that targeting HnRNPU could make cancer treatments more effective by promoting ferroptosis. In the future, this approach might help develop new therapies for colon cancer.

This summary was initially drafted using artificial intelligence, then revised and fact-checked by the author.

## Introduction

Ferroptosis is a newly discovered type of cell death mainly caused by excessive iron accumulation^1–3^. Increasing evidence links ferroptosis to a range of diseases, including cancer, ischemic organ injury and degenerative disorders^4–6^. Cancer cells with chemoresistance, KRAS mutations or high endogenous reactive oxygen species levels show enhanced susceptibility to ferroptosis inducers. Given these characteristics, targeting ferroptosis is a promising therapeutic strategy. However, the molecular mechanisms underlying ferroptosis remain unclear.

RNA-binding proteins (RBPs) regulate RNA processes, including splicing, stability, transport and translation^7^. Abnormal RBP expression contributes to cancer development and progression by modulating various biological functions, including chemoresistance, immune evasion, epithelial–mesenchymal transition, immune response, DNA repair and angiogenesis^8,9^. Recent studies have shown that RBPs play crucial roles in ferroptosis regulation. For instance, Wen et al. proposed that inhibition of the RBP RBMS1, which interacts with eukaryotic translation initiation factor 3, suppresses SLC7A11 translation, thereby reducing cystine uptake and promoting ferroptosis^10^. Jae et al. reported that silencing of poly(rC)-binding protein 1 inhibits BECN1 messenger RNA stability to enhance ferritinophagy and lipid peroxidation, which attenuating head and neck cancer susceptibility to ferroptosis^11^. However, the function of RBPs in ferroptosis regulation is not fully understood.

Heterogeneous nuclear ribonucleoprotein U (HnRNPU) is an RBP that belongs to the HnRNP family. HnRNPU performs diverse functions in RNA processing, including mRNA maturation, stabilization and translation. Abnormal expression of HnRNPU has been associated with cancer initiation and progression. Bo et al. reported that HnRNPU interacts with DEAD box helicase 5 to coordinate transcription and splicing in breast cancer cells to induce proliferation and migration^12^. Inhibition of HnRNPU enhances cisplatin sensitivity in bladder carcinoma cells by controlling the DNA damage repair pathways^13^. However, the role and molecular mechanisms of HnRNPU in the regulation of ferroptosis remain unknown.

In this study, we found that HnRNPU expression was upregulated in colon adenocarcinoma (COAD) tissues compared with normal colon tissues and is correlated with poor prognosis. HnRNPU knockdown inhibited proliferation and induced ferroptosis in COAD cells by inhibiting cystine uptake and glutathione (GSH) synthesis. Mechanistically, HnRNPU knockdown interrupted system xc^−^-mediated cystine uptake by destabilizing the mRNAs of SLC7A11 and SLC3A2. Our findings suggest a regulatory function of HnRNPU in ferroptosis within COAD, suggesting its potential as a therapeutic target to enhance the efficacy of ferroptosis-inducing agents.

## Materials and methods

### Cell culture and reagents

SW620, DLD-1, HCT-15, HCT116 and SW480 cells were obtained from the American Type Culture Collection (ATCC). Each cell line was cultured in Dulbecco’s modified Eagle medium or RPMI 1640 medium (ABSIN, cat. no. abs9560/abs9484). All cell culture media were supplemented with 1% penicillin–streptomycin (Epizyme Biotech, cat. no. CB010) and 10% fetal bovine serum (Procell, cat. no. 164210-50). These cell lines were maintained in a 5% CO_2_ humidified incubator at 37 °C. Liproxstatin-1 (HY-12726), RSL3 (HY-100218A), 3-methyladenine (3-MA; HY-19312), Z-VAD-FMK (HY-16658B) and necrostatin-1 (HY-15760) were purchased from MedChemExpress.

### Lentivirus transfection and stable cell line construction

Stable HnRNPU-knockdown cell lines, along with stable SLC3A2 and SLC7A11 overexpression cell lines, were generated via lentiviral transduction. Human Lenti-sh-HnRNPU-mNeonGreen, Lenti-oeSLC3A2-mScarlet, Lenti-oeSLC7A11-RFP and control groups were transfected (Beijing SyngenTech). Puromycin dihydrochloride (Beyotime, cat. no. ST551), G-418 disulfate (MedChemExpress, cat. no. HY-17561) and blasticidin (Yeasen, cat. no. 3513-03-9) were used to select successfully transfected cells. The nucleotide sequence of short hairpin RNA is provided in Supplementary Table 1.

### Cell total protein extraction and western blot analysis

Indicated COAD cell lines were lysed in RIPA buffer (Solarbio, cat. no. R0020) containing protease and phosphatase inhibitors (Solarbio, cat. no. P1260). Following centrifugation at 15,000 rpm for 20 min, the supernatants were collected. The protein concentrations were quantified, and the samples were denatured. Equal amounts of protein and a standard molecular weight marker (Solarbio, cat. no. RP1910) were separated on a 10% SDS–polyacrylamide gel electrophoresis gel and transferred to a polyvinylidene fluoride membrane. The membranes were blocked with skimmed milk (Biosharp, cat. no. BS102) and incubated with primary antibodies at 4 °C overnight. After three washes with TBST, membranes were incubated with secondary antibodies for 1 h and visualized using a chemiluminescent reagent (ZOMANBIO, cat. no. ZD310). All antibody information is presented in Supplementary Table 2.

### RNA isolation and RT–qPCR

RNA was extracted from cultured COAD cells using the RNAeasy Animal RNA Isolation Kit. The first complementary DNA strand was synthesized using the BeyoR Q cDNA Synthesis Kit (Beyotime, cat. no. R0027/D7190M). A 20 µl reaction mixture was prepared for each sample, containing 2 µl of each primer, 1 µl of cDNA template, 10 µl of SYBR Green PCR Mix (Beyotime, cat. no. D7262) and 7 µl of RNase-free water (Zoman, cat. no. ZS105). Gene expression levels were normalized to GAPDH and calculated using the 2^−ΔΔCT^ method. The following primers were used in this assay: GAPDH forward (F): 5′-GGAGCGAGATCCCTCCAAAAT and reverse (R): 5′-GGCTGTTGTCATACTTCTCATGG; SLC3A2 F:5′-TGAATGAGTTAGAGCCCGAGA and R:5′-GTCTTCCGCCACCTTGATCTT; SLC7A11 F:5′-TCTCCAAAGGAGGTTACCTGC and R:5′-AGACTCCCCTCAGTAAAGTGAC; GPX4 F:5′-GAGGCAAGACCGAAGTAAACTAC and R:5′- CCGAACTGGTTACACGGGAA.

### Cell cycle analysis

The indicated COAD cell lines were washed with 3 ml of cold phosphate-buffered saline (Solarbio, cat. no. P1022), centrifuged at 1,200 rpm for 4 min, and the supernatant was removed. Each tube was incubated overnight at −20 °C with 2 ml of 70% ethanol. On the following day, the cells were washed three times with phosphate-buffered saline and centrifuged at 1,400 rpm for 5 min to remove the ethanol. The cell pellets were then resuspended in 0.5 ml of PI/RNase staining buffer (BD, cat. no. 550825). After incubation at 37 °C for 30 min, the tubes were stored on ice and protected from light until analysis. Fluorescence intensity was measured using a flow cytometer.

### mRNA stability assay

A concentration of 6 mg/ml actinomycin D (MedChemExpress, cat. no. HY-17559) was used to assess mRNA stability in the SW620 and SW480 cell lines. The cells were collected at specific time points, and RNA was extracted from each sample for subsequent RT–qPCR analysis.

### RIP assay

RNA immunoprecipitation (RIP) assays were performed on cultured COAD cells using the PureBinding RNA Immunoprecipitation Kit (Geneseed, cat. no. P0101). According to the manufacturer’s instructions, lysates were immunoprecipitated with RIP buffer containing HnRNPU antibody coupled to magnetic beads. The RNA was then extracted from the RNA–protein complexes. The expression of SLC3A2 and SLC7A11 was analyzed using RT–qPCR. IgG served as the negative control, and the results were compared accordingly.

### Dual-luciferase assay

The full-length 5′ untranslated region (5′ UTR), coding DNA sequence (CDS) and 3′ UTR of human SLC3A2 mRNA were subcloned into the SV40-luc-5′ UTR/CDS/3′ UTR, respectively (Beijing SyngenTech). Similarly, the full-length 5′ UTR and CDS of SLC7A11 mRNA, along with the predicted 3′ UTR sequences 1 and 2 from the RBPsuite website, were subcloned into the SV40-luc-5′ UTR/CDS/3′ UTR#1/3′ UTR#2, respectively (Shanghai Genechem). The cells in the negative control group were cotransfected with the SV40-luc-MCS (pmirGLO). A Renilla luciferase vector was used to normalize transfection efficiency in each group. SW480 cells were seeded into 24-well plates and divided into four groups, each with three replicates. Transfection with the 5′ UTR, CDS and 3′ UTR was performed using the Lipofectamine 3000 Reagent (Thermo Fisher Scientific, cat. no. L3000015). As per the manufacturer’s instructions, the cells were collected 48 h post transfection, and luciferase activity was monitored using the Dual-Luciferase Reporter Assay System (Promega, cat. no. E1910).

### Tissue microarray and COAD fresh samples

A COAD tissue microarray was purchased from Shanghai Outdo Biotech Company (HColA180Su19) and included 178 clinical samples with detailed pathological and survival information. Fresh cancer tissues were obtained from ten patients with primary COAD. The samples were surgically resected at the Affiliated Hospital of Yanbian University (Yanji, China), without preoperative chemotherapy, radiotherapy or other tumor-specific treatments. Each sample consisted of one tumor specimen and a corresponding adjacent normal tissue specimen. A pathologist conducted postoperative pathological confirmation. The Medical Ethics Committee of the Affiliated Hospital of Yanbian University approved this study (approval no. 2025247).

### Immunofluorescence staining

SW620 and SW480 cells were seeded into six-well plates at a confluency of 50–70%. On the following day, transfected cell colonies were fixed with 4% paraformaldehyde (Beyotime, cat. no. P0099) for 45 min, permeabilized with Triton X-100 (Beyotime, cat. no. ST1723) for 3 min and blocked with 5% BSA (Solarbio, cat. no. A8020) for at least 70 min. The cells were then incubated with primary antibodies (SLC7A11 and SLC3A2) at 4 °C overnight. The next day, SW620 and SW480 cells were incubated with secondary antibodies in the dark for 1 h and counterstained with 4′,6-diamidino-2-phenylindole. The images were acquired using an OLYMPUS FV3000 confocal laser scanning microscope, and ImageJ software was used to analyze the images.

### Immunohistochemical staining

Paraffin-embedded sections were deparaffinized, rehydrated and subjected to antigen retrieval using a buffer (Beyotime, cat. no. P0084). Primary antibodies against HnRNPU, Ki-67, 4-HNE, SLC7A11 and SLC3A2 were incubated overnight at 4 °C, followed by incubation with secondary antibodies. The DAB working solution was then added to each section, and the color development was observed under a microscope. All tissue sections were counterstained with hematoxylin (Solarbio, cat. no. G1080) and observed. Immunohistochemistry (IHC) staining intensity was classified into four semiquantitative categories: 0 (negative), 1 (weak), 2 (moderate) and 3 (strong). These intensity scores were multiplied by the staining area grade (ranging from 0 to 4, corresponding to 0–100%) to calculate the IHC score for each sample.

### Xenograft model

All mice were obtained from Vital River Laboratory Animal Technology and maintained under specific pathogen-free conditions. For the subcutaneous tumor model, 6 × 10^6^ SW620 cells were injected into the right armpit of BALB/c nude mice. Tumor diameter, volume and body weight were measured every 3 days. After 4 weeks, the animals were killed, and the subcutaneous tumors were removed. The tumor volume was calculated using the following formula: tumor volume = (long diameter) × (short diameter)^2^ × 0.5. All experiments were approved by the Laboratory Animal Ethics Committee Yanbian University.

### Bioinformatics data and statistical analysis

Bioinformatics analyses were conducted using publicly available databases and automated statistical tools, including the Gene Expression Omnibus (GEO) (GSE68468 and GSE81582), The Cancer Genome Atlas (TCGA) database, TIMER 2.0, UALCAN, FerrDb, STRING, RBPsuite and XIANTAO platform^14–18^. The data analyses were performed using GraphPad Prism (version 9.0; GraphPad Software), SPSS (version 26.0; IBM SPSS) and ImageJ software (version 1.52a; National Institutes of Health). The group differences were analyzed using an analysis of variance, Student’s *t*-test and Pearson’s analysis. All data are expressed as mean ± standard deviation, and experiments were performed in triplicate or more.

## Results

### HnRNPU is overexpressed in COAD tissues and correlates with poor prognosis

**Fig. 1:**
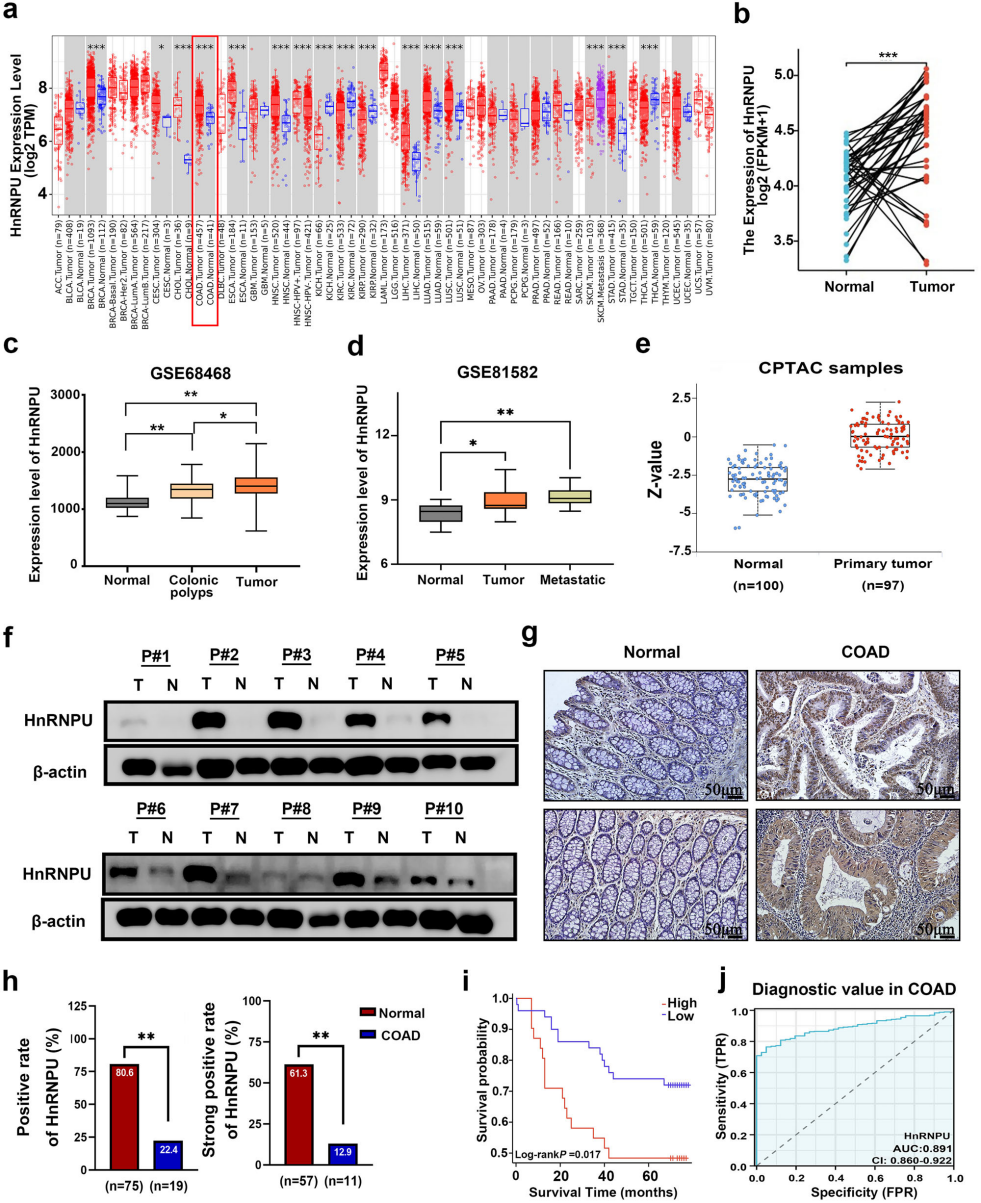
HnRNPU is overexpressed in COAD tissues and correlates with poor prognosis. **a** The expression of HnRNPU mRNA in normal and COAD tissues was analyzed using TIMER 2.0 database. **b** The mRNA expression levels of HnRNPU in COAD tissues and paired adjacent normal tissues were obtained from the TCGA database. **c**, **d** The mRNA expression levels of HnRNPU in normal tissues, colonic polyps tissues, primary colorectal cancer (CRC) tissues and metastatic CRC tissues were analyzed using the GEO databases (GSE68468 and GSE81582). **e** The HnRNPU protein expression in normal and CRC tissues was analyzed using the CPTAC database. **f** The protein expression levels of HnRNPU in ten paired samples of COAD tissues and adjacent nontumor tissues were detected using western blot analysis. β-actin was included as an internal control. **g** A representative image of HnRNPU protein expression in normal and COAD tissues were detected using IHC. Scale bar, 50 μm. **h** The positive or strong positive rate of HnRNPU protein in COAD and normal colon tissues was analyzed. **i** Kaplan–Meier analysis curves show the correlation between HnRNPU expression and overall survival rate of patients with COAD. **j** ROC curves were used to determine the diagnostic value of HnRNPU in COAD using the XIANTAO platform. *P* value significant codes: *P < 0.05; **P < 0.01; ***P < 0.001.

To elucidate the expression status of HnRNPU in COAD, mRNA expression levels were analyzed in pan-cancer cells using the TIMER 2.0 database. The results demonstrated significant upregulation of HnRNPU in various cancer types, including BRCA, CHOL, COAD and ESCA (Fig. 1a). Prediction of HnRNPU expression using the XIANTAO platform confirmed the high expression in COAD tissues (Fig. 1b). These findings were validated using GEO database analysis, which showed the upregulated expression of HnRNPU in colon polyps, primary colon cancer and metastatic colon cancer compared with normal colon tissues (Fig. 1c-d). In addition, UALCAN database analysis showed that HnRNPU protein expression was increased in colon cancer tissues (Fig. 1e and Supplementary Fig. 1a).

HnRNPU protein expression was detected in ten paired COAD tissues and their corresponding adjacent normal colon tissues. We observed that the HnRNPU protein was upregulated in eight of the ten COAD tissues (Fig. 1f). To further confirm the protein expression status and clinical relevance of HnRNPU in COAD, IHC was performed using a tissue microarray containing 93 COAD tissues and 85 matched adjacent nontumor tissues (Fig. 1g). HnRNPU expression was higher in COAD tissues (80.6%, 76/93) than in adjacent nontumor tissues (22.4%, 19/85, *P* < 0.01) (Fig. 1h). Kaplan–Meier analysis showed that patients with high HnRNPU expression experienced shorter overall survival than those with low HnRNPU expression (*P* = 0.017) (Fig. 1i). Moreover, Cox regression analysis identified high HnRNPU expression (*P* = 0.006) and histological grade (*P* = 0.041) as independent prognostic factors for patients with COAD (Supplementary Table 3). Notably, receiver operating characteristic curve (ROC) analysis showed that HnRNPU had good accuracy in predicting the prognosis of COAD (area under the curve of 0.891) (Fig. 1j). These results indicate that HnRNPU expression is increased in COAD and may serve as a predictor of poor prognosis.

### HnRNPU knockdown inhibits cell proliferation and induces cell cycle arrest

**Fig. 2:**
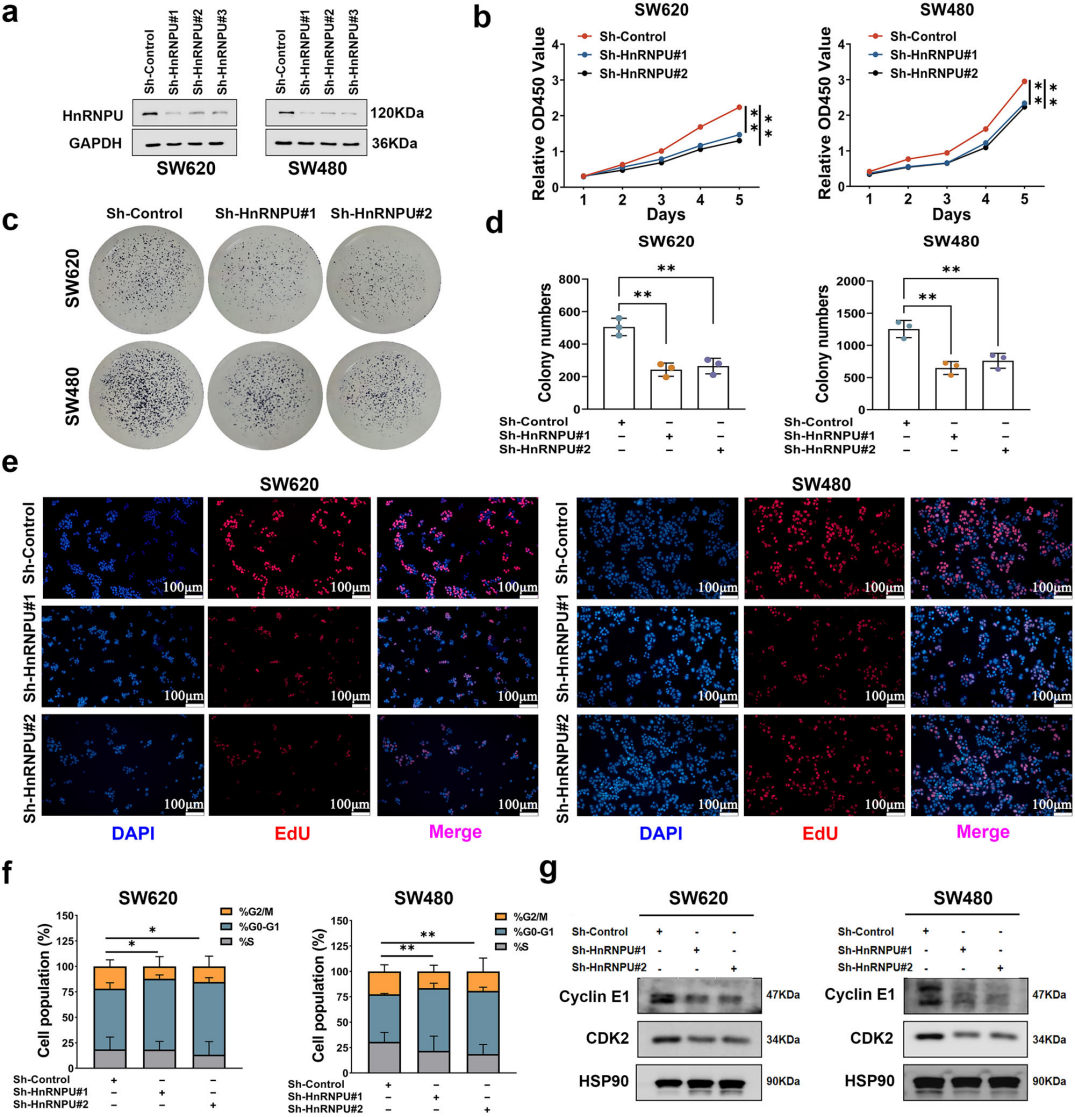
HnRNPU knockdown inhibits cell proliferation and induces cell cycle arrest. **a** Western blot analysis were performed to detect HnRNPU expression in SW620 and SW480 cells with sh-HnRNPU transfection. GAPDH was included as an internal control. **b** Cell viability was determined using CCK-8 in SW620 and SW480 cells with HnRNPU knockdown. **c**, **d** The effects of HnRNPU knockdown on cell proliferation were determined using the colony formation assay. **e** The effects of HnRNPU knockdown on DNA replication were determined using the EdU staining. Scale bar, 100 μm. **f** The cell cycle distributions of SW620 and SW480 cells with HnRNPU knockdown were detected using flow cytometry. **g** Cell-cycle-related proteins were detected using western blot analysis in SW620 and SW480 cells with HnRNPU knockdown. *P* value significant codes: *P < 0.05; **P < 0.01.

To investigate the biological function of HnRNPU in COAD, we examined its expression in various colon cancer cell lines (Supplementary Fig. 1b). Three independent short hairpin RNAs targeting HnRNPU were transfected into SW620 and SW480 cells to generate HnRNPU-knockdown cells, and their efficacy was determined using western blot (Fig. 2a). We explored the role of HnRNPU in cell proliferation using CCK-8, EdU and colony formation assays, all of which showed that HnRNPU knockdown inhibited COAD cell proliferation (Fig. 2b–e and Supplementary Fig. 1c). HnRNPU has been reported to bind to the CDK2 gene locus and regulate cell cycle distribution in hepatocellular carcinoma^19^. Thus, we investigated whether HnRNPU modulates cell cycle progression in COAD cells. As shown in Fig. 2f, HnRNPU knockdown induced G0/G1 phase arrest and decreased the expression of cell-cycle-related proteins cyclin E1 and CDK2 (Fig. 2g and Supplementary Fig. 1d). These findings demonstrate that HnRNPU regulates COAD cell proliferation and cell cycle progression.

### HnRNPU knockdown induces ferroptosis in COAD by inhibiting system xc^−^/GSH/GPX4 axis

**Fig. 3:**
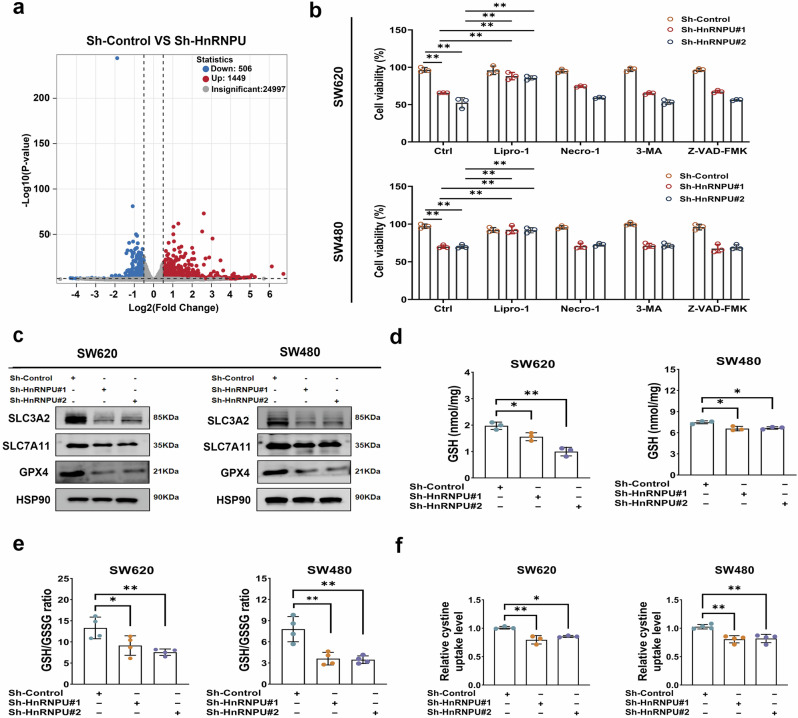
HnRNPU knockdown induces ferroptosis in COAD by inhibiting system xc^−^/GSH/GPX4 axis. **a** The differentially expressed genes were identified using RNA sequencing in SW620 cells with HnRNPU knockdown. Significantly upregulated and downregulated proteins are highlighted in red and blue, respectively. The curve is derived with a false discovery rate of 0.05. **b** Cell viability was determined using CCK-8 in SW620 and SW480 cells with HnRNPU knockdown in the absence or presence of liproxstatin-1 (1 μM), necrosulfonamide (1 μM), Z-VAD-FMK (10 μM) or 3-methyladenine (200 μM) for 24 h. **c** The expression of GPX4, SLC3A2 and SLC7A11 was detected in HnRNPU-knockdown cells by using western blot analysis. HSP90 was included as an internal control. **d**, **e** The level of GSH and the ratio of GSH/GSSG were determined in HnRNPU-knockdown cells. **f** Cystine uptake capacity was determined in HnRNPU-knockdown cells. *P* value significant codes: *P < 0.05; **P < 0.01.

To understand the biological function of HnRNPU, RNA-sequencing analysis was performed on HnRNPU-knockdown cells. A total of 1,955 differentially expressed genes were identified, including 1,449 upregulated and 506 downregulated genes (Fig. 3a). A Kyoto Encyclopedia of Genes and Genomes analysis revealed that the differentially expressed genes were related to phagosomes, ferroptosis, cytokine-cytokine receptor interactions and cell adhesion molecules (Supplementary Fig. 2a). Based on these observations, we hypothesized that HnRNPU knockdown may partially inhibit cell growth by triggering ferroptosis. To confirm this hypothesis, HnRNPU-deleted COAD cells were treated with different cell death inhibitors, including ferroptosis inhibitor liproxstatin-1, apoptosis inhibitor Z-VAD-FMK, the autophagy inhibitor 3-MA and necroptosis inhibitor necrostatin-1. Notably, treatment with liproxstatin-1 partially reversed the growth inhibition caused by HnRNPU deletion, whereas 3-MA, necrostatin-1 and Z-VAD-FMK had no effect (Fig. 3b). These results suggest that HnRNPU knockdown inhibited cell growth by eliciting ferroptosis.

We explored the function of HnRNPU in triggering ferroptosis. Western blot analysis revealed decreased expression of SLC3A2, SLC7A11 and GPX4 (Fig. 3c and Supplementary Fig. 2b), while 4-HNE, a product of lipid peroxidation, was increased in HnRNPU-knockdown cells (Supplementary Fig. 2c,d). In addition, HnRNPU knockdown decreased GSH levels, cystine uptake capacity and the GSH/GSSG ratio in COAD cells (Fig. 3d–f). In addition, IF staining further confirmed the downregulation of SLC3A2 and SLC7A11 (Supplementary Fig. 3a–b). However, levels of the iron regulatory proteins FTH1 and TFRC, as well as intracellular ferrous iron, showed no change (Supplementary Fig. 4a–d). Overall, these results demonstrate that knockdown of HnRNPU induces ferroptosis by inhibiting GSH synthesis through suppression of the xc^−^/GSH/GPX4 axis.

### HnRNPU knockdown enhances the sensitivity of COAD cells to ferroptosis

**Fig. 4:**
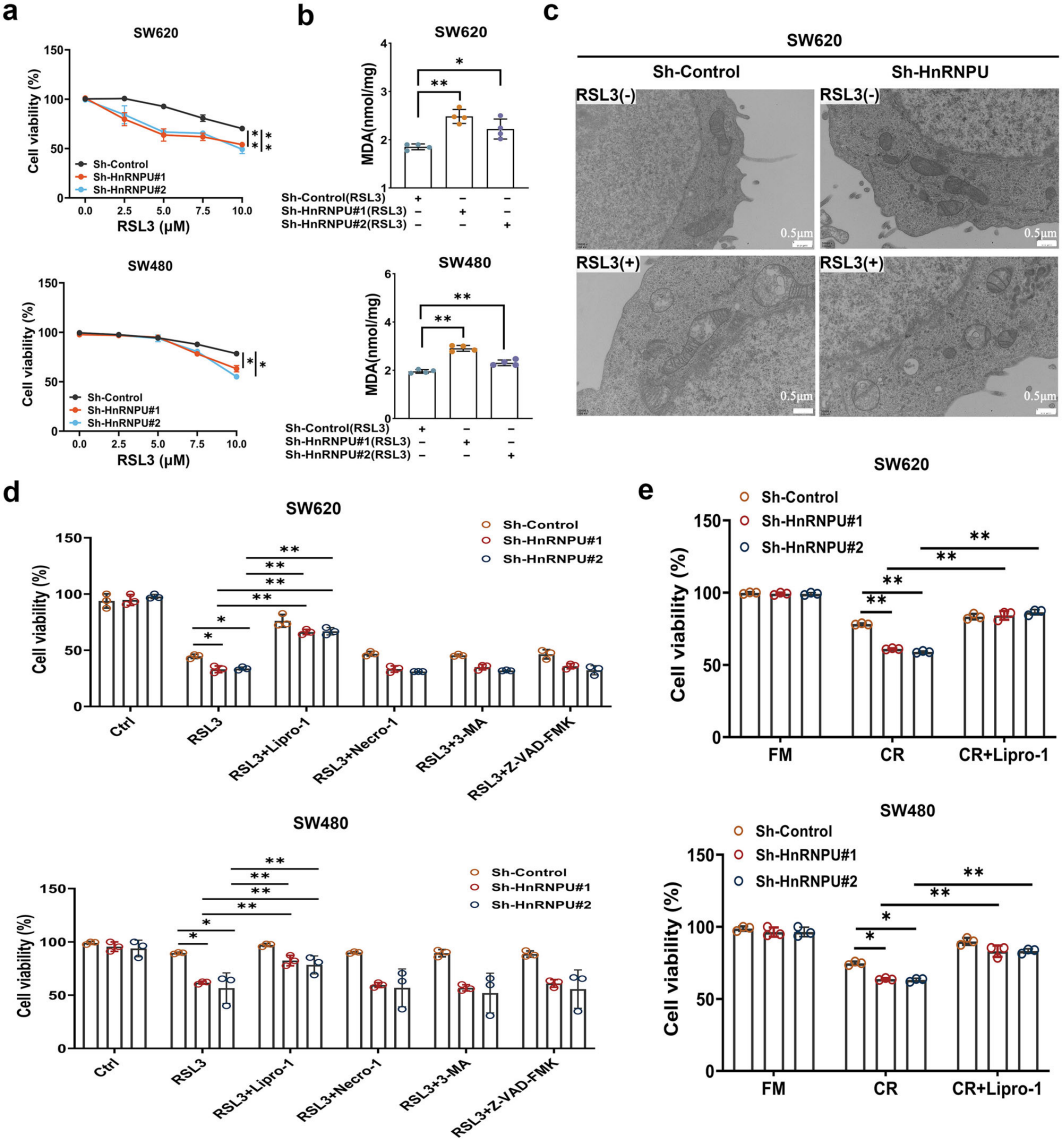
HnRNPU knockdown enhances the sensitivity of COAD cells to ferroptosis. **a** Cell viability was determined using CCK-8 in SW620 and SW480 cells expressing sh-con or sh-HnRNPU treated with different concentrations of RSL3 for 24 h. **b** MDA assay kit was used to detect the level of MDA treated with RSL3 (SW620:RSL3 5 μM; SW480:RSL3 7.5 μM). **c** An electron microscopy technique was applied to determine the changes occurring in the cellular mitochondria to assess ferroptosis. Scale bar, 0.5 μm. **d** The cell viability was determined using CCK-8 in SW620 and SW480 cells expressing sh-con or sh-HnRNPU treated with RSL3 in the absence or presence of liproxstatin-1 (1 μM), necrosulfonamide (1 μM), Z-VAD-FMK (10 μM) or 3-methyladenine (200 μM) for 24 h (SW620:RSL3 5 μM; SW480:RSL3 7.5 μM). **e** Cell viability was determined using CCK-8 in SW620 and SW480 cells expressing sh-con or sh-HnRNPU cultured under cystine restriction for 24 h alone or in combination with liproxstatin-1 as indicated is shown. *P* value significant codes: *P < 0.05; **P < 0.01.

Given that HnRNPU knockdown inhibits GSH synthesis, we hypothesized that HnRNPU knockdown may promote the sensitivity of COAD cells to ferroptosis inducers. To confirm this hypothesis, we subjected the established SW620 and SW480 cells to the ferroptosis inducer RSL3 and assessed the activity of the COAD cell lines using CCK-8 assays. As predicted, the HnRNPU depletion facilitated RSL3-induced ferroptosis (Fig. 4a), as indicated by increased malondialdehyde (MDA) levels (Fig. 4b). Transmission electron microscopy revealed that upon treatment with RSL3, HnRNPU knockdown exhibited significantly enhanced ferroptosis-specific mitochondrial changes, such as mitochondrial swelling and shrunken mitochondrial cristae (Fig. 4c). Consistently, HnRNPU-depleted COAD cells exhibited robust resistance to RSL3-induced ferroptosis when treated with liproxstatin-1 but not when treated with 3-MA, necrostatin-1 or Z-VAD-FMK (Fig. 4d). Similar results were observed when using light microscopy, sh-HnRNPU in combination with RSL3 resulted in cell shrinkage and morphological disruption, which were reversed by liproxstatin-1 (Supplementary Fig. 5a). Interestingly, similar results were observed using CCK-8 assays and light microscopy, where sh-HnRNPU in combination with cystine-restricted medium resulted in low cell survival, which was rescued by liproxstatin-1 (Fig. 4e and Supplementary Fig. 5b). Collectively, these data indicate that HnRNPU suppression sensitizes COAD cells to RSL3- or cystine-restricted mediated ferroptosis.

### HnRNPU promotes SLC7A11 and SLC3A2 mRNA stability in COAD cells

**Fig. 5:**
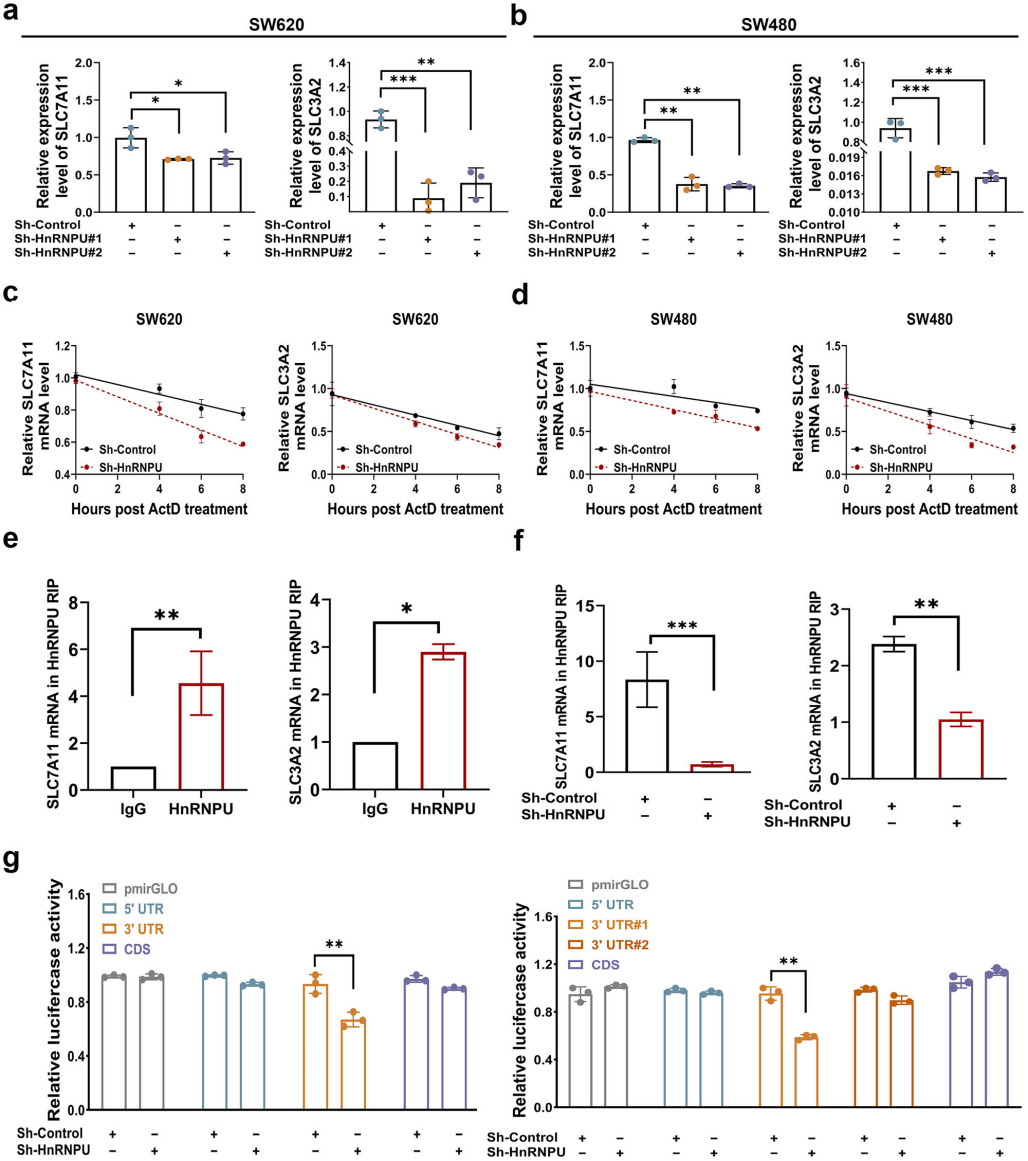
HnRNPU promotes SLC7A11 and SLC3A2 mRNA stability in COAD cells. **a**, **b** RT–qPCR was performed to detect mRNA expression of SLC7A11 and SLC3A2 in SW620 (**a**) and SW480 (**b**) cells with HnRNPU-knockdown. **c**, **d** RT–qPCR was performed to evaluate the mRNA stability of SLC7A11 and SLC3A2 after adding actinomycin D in SW620 (**c**) and SW480 (**d**) cells with HnRNPU-knockdown. **e** RIP assay was used to analyzed the interaction between HnRNPU and SLC7A11, along with SLC3A2 in SW620 cells. **f** RIP assay was used to analyze the interaction between HnRNPU and SLC7A11, along with SLC3A2 in HnRNPU-knockdown cells. **g** Dual-luciferase reporter assays were used with SLC3A2-5′ UTR/CDS/3′ UTR/ and SLC7A11-5′ UTR/CDS/3′ UTR#1/3′ UTR#2. *P* value significant codes: *P < 0.05; **P < 0.01, ***P < 0.001.

To further explore the molecular mechanism of the HnRNPU-mediated inhibition of system xc^−^, we measured the mRNA levels of SLC3A2 and SLC7A11 in HnRNPU-knockdown cells. The mRNA levels of both decreased, whereas GPX4 showed no change (Fig. 5a–b and Supplementary Fig. 6a). HnRNPU has been reported to specifically bind to the 3′ UTR of mRNA and subsequently promote RNA stability^20^. The STRING database also revealed that HnRNPU-interacting proteins were functionally clustered into the RNA-processing subgroup, which included RNA splicing, transport and stabilization (Supplementary Fig. 6b). Based on these observations, we hypothesized that HnRNPU affects the mRNA stability of SLC3A2 and SLC7A11. Consistent with our hypothesis, the actinomycin D assay showed that the half-life of SLC3A2 and SLC7A11 mRNA was shorter in HnRNPU-knockdown cells, indicating that HnRNPU can regulate the mRNA stability of SLC3A2 and SLC7A11 (Fig. 5c, d). Then, we investigated whether HnRNPU binds to SLC3A2 and SLC7A11 mRNA. RBPsuite, an online website, predicted potential binding sites between HnRNPU protein and SLC7A11 mRNA, along with SLC3A2 (Supplementary Fig. 6c). The RNP-IP assay showed that SLC3A2 and SLC7A11 mRNA were enriched in the materials pulled down by the HnRNPU antibody, whereas the sh-HnRNPU groups showed a significant decrease in SLC3A2 and SLC7A11 mRNA (Fig. 5e, f). We then constructed luciferase reporter plasmids containing the 5′ UTR, 3′ UTR and CDS of SLC3A2 and the 5′ UTR, 3′ UTR#1, 3′ UTR#2 and CDS of SLC7A11 and transfected them into SW480 cells to identify the precise binding site (Supplementary Fig. 6d). The results showed that HnRNPU directly interacts with the 3′ UTR of SLC3A2 and SLC7A11 (Fig. 5g). Taken together, these findings demonstrate that HnRNPU regulates SLC3A2 and SLC7A11 mRNA stability by directly binding with 3′ UTR.

### HnRNPU regulates system xc^−^ -mediated cystine uptake and enhances ferroptosis in COAD

**Fig. 6:**
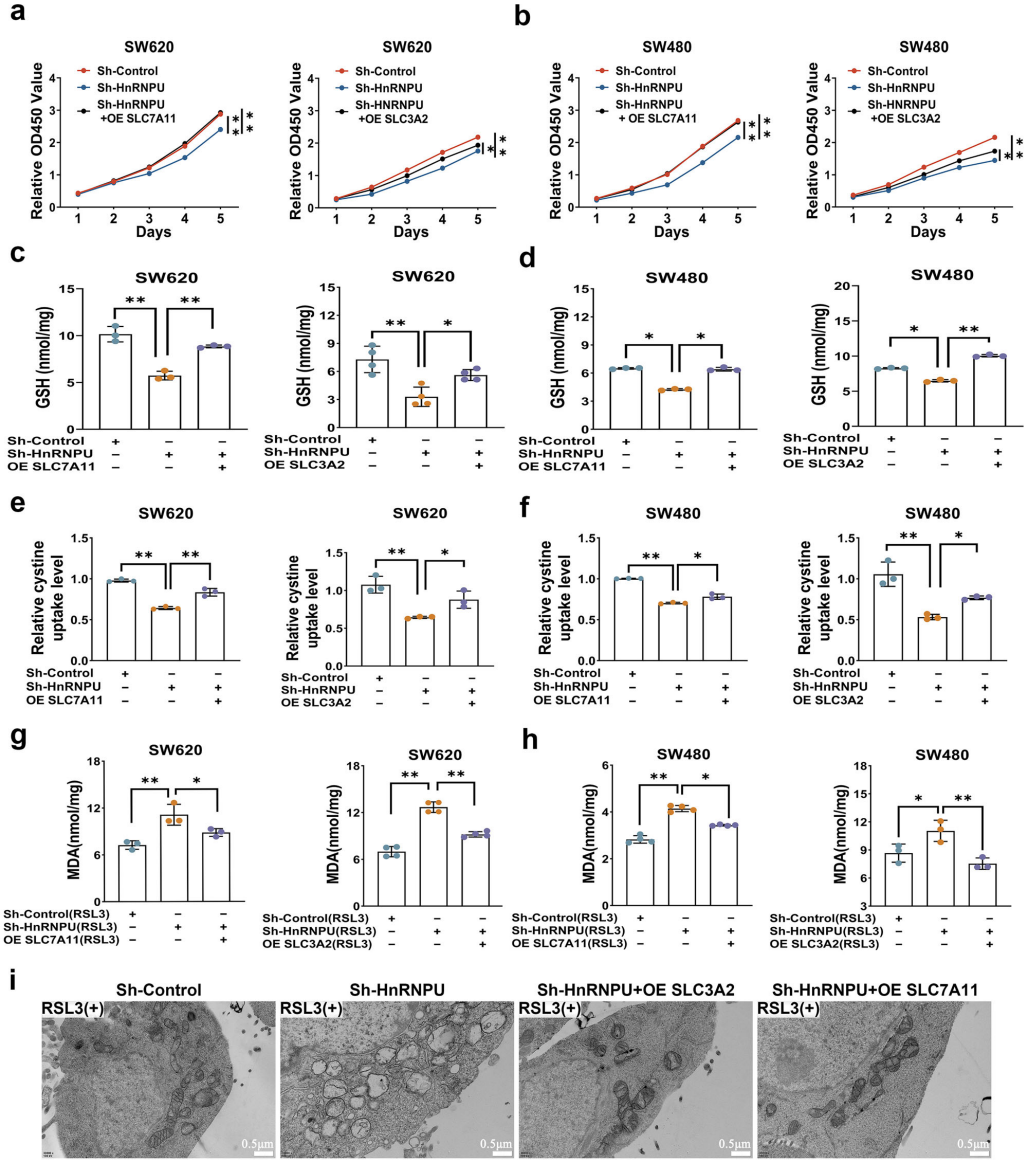
HnRNPU regulates system xc^−^ -mediated cystine uptake and enhances ferroptosis in COAD. **a**, **b** Cell viability was determined using CCK-8 in HnRNPU-knockdown SW620 (**a**) and SW480 (**b**) cells with or without SLC3A2 or SLC7A11 overexpression. **c**, **d** GSH level was detected in HnRNPU-knockdown SW620 (**c**) and SW480 (**d**) cells with or without SLC3A2 or SLC7A11 overexpression. **e**, **f** Cystine uptake was detected in HnRNPU-knockdown SW620 (**e**) and SW480 (**f**) cells with or without SLC3A2 or SLC7A11 overexpression. **g**, **h** MDA was detected in HnRNPU-knockdown SW620 (**g**) and SW480 (**h**) cells with or without SLC3A2 or SLC7A11 overexpression, under RSL3 treatment. **i** An electron microscopy technique was applied to determine the changes occurring in the cellular mitochondria in HnRNPU-knockdown cells with or without SLC3A2 or SLC7A11 overexpression with RSL3 treatment. Scale bar, 0.5 μm. *P* value significant codes: *P < 0.05; **P < 0.01.

We examined whether the downregulation of system xc^−^ contributes to HnRNPU-depletion induced suppression of cystine uptake and GSH synthesis. SLC3A2 or SLC7A11 overexpression plasmid were separately transfected into HnRNPU-knockdown cells, western blot analysis confirmed the efficiency of the transfections (Supplementary Fig. 7a). As expected, SLC3A2 or SLC7A11 overexpression restored the proliferation of COAD cells inhibited by HnRNPU knockdown, as determined by CCK-8 and colony formation assays (Fig. 6a, b and Supplementary Fig. 7b, c). Enhanced expression of these genes also increased cystine uptake and GSH levels while reducing MDA levels in HnRNPU-knockdown cells (Fig. 6c–h). TEM analysis revealed that the altered mitochondrial ultrastructure in sh-HnRNPU cells treated with RSL3 was restored by SLC3A2 and SLC7A11 overexpression (Fig. 6i). These results indicate that HnRNPU knockdown inhibits proliferation, GSH synthesis and cystine uptake by regulating system xc^−^.

### Expression of HnRNPU and system xc^−^ positively correlates in COAD tissues

**Fig. 7:**
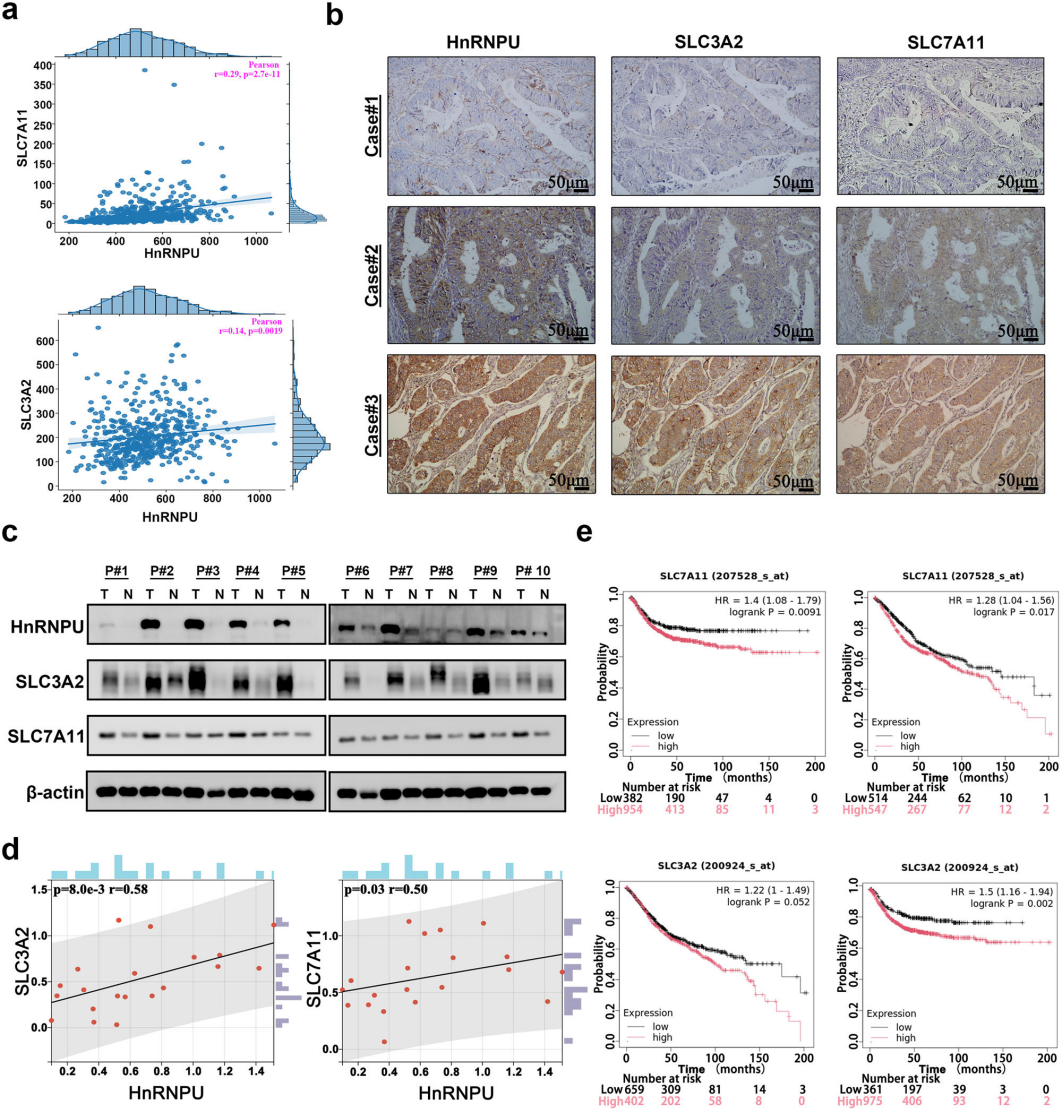
Expression of HnRNPU and system xc^−^ was positively correlated in COAD tissues. **a** The relationship between mRNA expression of HnRNPU and system xc^−^ (SLC3A2/SLC7A11) was analyzed using the FerrDb database. **b** An IHC staining of HnRNPU and system xc^−^ in COAD tissues. **c**, **d** A western blot analysis (**c**) confirmed the HnRNPU and system xc^−^ in COAD tissues. β-actin was included as an internal control. A Spearman correlation analysis (**d**) was performed to analyze the correlation. **e** The relationship between system xc^−^ mRNA expression levels and survival of patients with colon cancer were determined using the Kaplan–Meier analysis.

Given that HnRNPU can regulates SLC3A2 and SLC7A11 expression in COAD cells, we sought to confirm this finding in COAD tissues. The FerrDb database showed a positive correlation between HnRNPU and both SLC3A2 and SLC7A11 (Fig. 7a). IHC and western blot analysis were performed to evaluate the correlation between HnRNPU and SLC3A2 and SLC7A11 in ten COAD samples and matched normal tissues. The results showed significantly higher expression of SLC3A2 and SLC7A11 in COAD tissues compared with normal tissues. A significant correlation was observed between HnRNPU expression and both SLC7A11 and SLC3A2 levels (Fig. 7b–d). Furthermore, high SLC7A11 expression was associated with significantly worse overall survival (OS) (*P* = 0.017) and recurrence-free survival (*P* = 0.0091). Meanwhile, high SLC3A2 expression was significantly correlated with poorer recurrence-free survival (*P* = 0.002), the association with OS (*P* = 0.052) approached but did not reach statistical significance, potentially owing to the limited sample size (Fig. 7e). These results demonstrate a positive correlation between HnRNPU expression and SLC7A11 and SLC3A2 in COAD.

### HnRNPU knockdown inhibits cell growth and induces ferroptosis in vivo

**Fig. 8:**
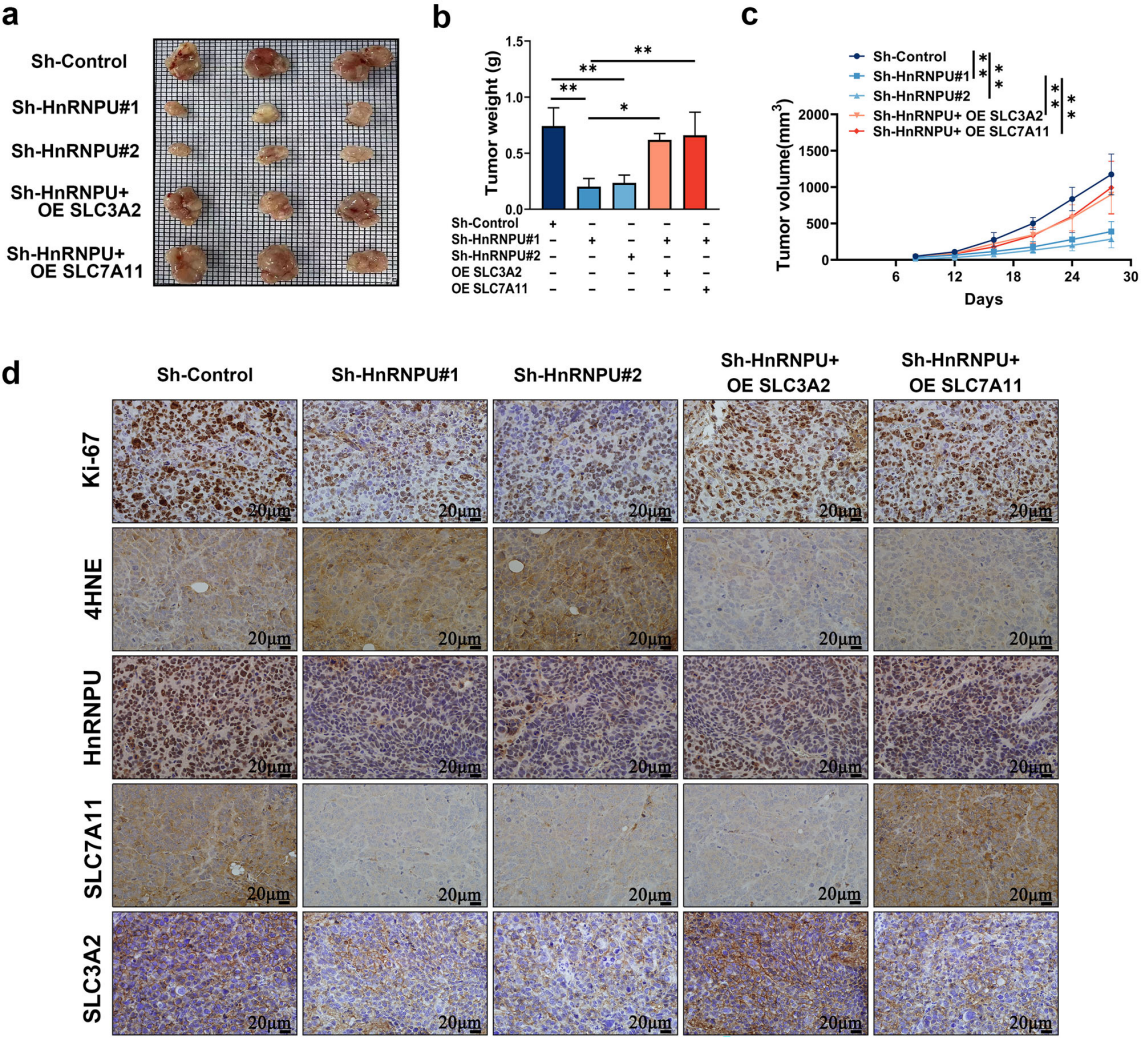
HnRNPU knockdown inhibits cell growth and induces ferroptosis in vivo. **a** Representative tumor image of xenograft mouse (*n* = 3). **b**, **c** The tumor weights (**b**) and volumes (**c**) were measured. **d** The expression patterns of Ki-67, 4-HNE, HnRNPU, SLC7A11 and SLC3A2 were assessed using IHC staining in xenograft mouse tissues. Scale bar, 50 μm. *P* value significant codes: *P < 0.05; **P < 0.01.

To further evaluate the effects of HnRNPU on tumorigenesis in vivo, a subcutaneous xenograft model was established in BALB/c nude mice. Subcutaneous tumors in the HnRNPU knockdown group were significantly smaller than those in the sh-control group, and this effect was mitigated by SLC3A2 or SLC7A11 overexpression (Fig. 8a, b). In addition, tumor volume was significantly decreased in the HnRNPU-knockdown group, and this effect was reversed by SLC3A2 and SLC7A11 overexpression (Fig. 8c). Moreover, IHC staining showed that Ki-67 expression was decreased in the HnRNPU knockdown group, whereas 4-HNE expression was increased; these observations were reversed by SLC3A2 and SLC7A11 overexpression (Fig. 8d). These results indicate that HnRNPU knockdown inhibits proliferation and promotes ferroptosis by regulating SLC3A2 and SLC7A11 in vivo.

## Discussion

RBP-mediated gene regulation drives various biological processes in cancer, including proliferation, chemoresistance, invasion, autophagy and angiogenesis. Recent studies, including our own, have demonstrated that RBPs play crucial roles in regulating ferroptosis in cancer. For example, Zhang et al. reported that the RBP ZFP36/TTP protects cancer cells from ferroptosis by regulating autophagy through binding to ATG16L1 mRNA^21^. Sun et al. found that the RBP NKAP enhances resistance to ferroptosis in glioblastoma by promoting SLC7A11 mRNA splicing^22^. Our previous study showed that RSL1D1 regulates ferroptosis in COAD cells by binding to FTH1 mRNA^23^. These observations demonstrate the important role of RBPs in modulating ferroptosis and highlight the potential of targeting these proteins for cancer treatment. In the present study, we identified the RBP HnRNPU as frequently upregulated in COAD tissues. High HnRNPU expression correlated with poor prognosis in patients with COAD. HnRNPU knockdown inhibited proliferation, cystine uptake and GSH synthesis by decreasing SLC7A11 and SLC3A2 expression, thereby inducing ferroptosis in COAD cells. Mechanistically, HnRNPU directly bound to the 3′ UTRs of SLC3A2 and SLC7A11 mRNAs, enhancing their stability.

In addition to intracellular iron accumulation, impairment of antioxidant systems, such as the system xc^−^/GSH/GPX4 axis also triggers ferroptosis. System xc^−^ is a sodium-independent inverse transporter consisting of two subunits linked by disulfide bonds: the heavy-chain subunit (SLC3A2) and the light-chain subunit (SLC7A11)^24–26^. SLC3A2 functions as a chaperone that stabilizes SLC7A11, whereas SLC7A11 mediates cystine/glutamate reverse transporter^27^. System xc^−^ takes up cystine to synthesize GSH, a well-known cofactor for GPX4, which protects cells from lipid peroxidation^4,28^. Our findings showed that HnRNPU depletion downregulated SLC3A2 and SLC7A11, inhibiting cystine uptake and GSH synthesis. This led to lipid peroxidation and ultimately induced ferroptotic cell death. Decreased GSH levels improved sensitivity to RSL3 and cystine-deprivation-induced ferroptosis.

An important finding of our study was the identification of the mechanism by which HnRNPU mediates the inhibition of SLC7A11 and SLC3A2. As an RBP, HnRNPU is involved in various RNA processes, including alternative splicing, translation and mRNA stability. For example, Han et al. reported that HnRNPU binds to MCM10 pre-mRNA and drives RNA splicing in triple-negative breast cancer^12^. In addition, Wang et al. showed that HnRNPU regulates the mRNA translation of MDM2 and RAN in myeloma cells, which subsequently confers resistance to selinexor^29^. Qu et al. reported that HnRNPU regulates BACE1 expression by stabilizing its mRNA^30^. In agreement with these findings, our study revealed that HnRNPU stabilizes the mRNAs of SLC3A2 and SLC7A11, thereby regulating their protein expression. Notably, HnRNPU depletion downregulated the protein expression of GPX4 but not its mRNA expression, suggesting an indirect regulatory mechanism. We believe that this mechanism is related to cystine uptake. Previous studies have shown that cystine not only promotes GSH synthesis but also partially enhances GPX4 protein synthesis via the Rag-mTORC1-4EBP axis^31^. Thus, we hypothesized that GPX4 protein expression, which was reduced by HnRNPU depletion, was partially dependent on reduced cysteine levels. However, further studies are needed to confirm this hypothesis.

**Fig. 9:**
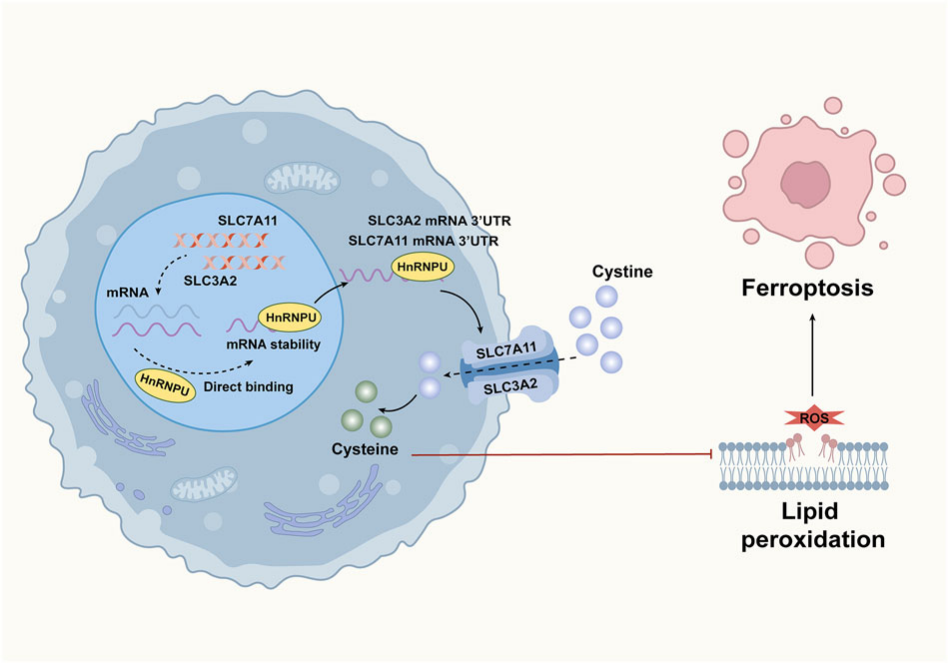
Schematic illustration of HnRNPU-mediated ferroptosis inhibition. This model graphically summarizes that HnRNPU binds directly to the 3′ UTRs of SLC7A11 and SLC3A2 mRNAs to enhance their stability, leading to increased expression of these subunits of the cystine/glutamate transporter. This upregulation promotes cystine uptake, suppresses lipid peroxidation and consequently inhibits ferroptosis.

In conclusion, our results emphasize the prominent regulatory mechanism of the HnRNPU–xCT axis. HnRNPU depletion alleviates the expression of CDK2 and cyclin E1, leading to cell cycle arrest. By contrast, HnRNPU acts as a ferroptosis inhibitor by increasing the mRNA stability of critical ferroptosis defense genes such as SLC3A2 and SLC7A11 (Fig. 9). Collectively, our study presents a novel perspective on the oncogenic potential of HnRNPU and its associated molecular mechanisms. In particular, HnRNPU may serve as a potential molecular target and provide a molecular basis for COAD treatment.

## Supplementary information

Supplementary Information

## Data Availability

The data that support the findings of this study are available on request from the corresponding author. The data are not publicly available due to privacy or ethical restrictions.
