# Supplementary material for: RNA-binding protein HnRNPU regulates proliferation and ferroptosis in colon adenocarcinoma by stabilizing the mRNA of system xc−
Source: Exp Mol Med. 2025 Nov 28;57(11):2686–98. doi: 10.1038/s12276-025-01569-z (PMC12686487; doi:10.1038/s12276-025-01569-z)
Supplement: Supplementary file 1 — Supplementary Information [file 12276_2025_1569_MOESM1_ESM.pdf]

Supplementary Information for

RNA-binding protein HnRNPU regulates proliferation and ferroptosis in colon adenocarcinoma by stabilizing the mRNA of system xc-

This file includes:

Supplementary Materials and methods

Supplementary Tables 1 to 3

Supplementary Figures 1 to 7

Supplementary Materials and methods

Cell viability analysis and colony formation assay

For the cell viability assay, 5 × 10^3 SW620 and SW480 cells were seeded into 96-well plates. Cell proliferation was assessed using the CCK-8 reagent (cat. no. IV08-500; Invigentech, USA). After treating the cells with indicated concentrations of inhibitor for designated durations, 10 μL of CCK-8 reagent was added to each well. Cells were incubated at 37 °C for 2 h, and absorbance at 450 nm was measured.

For the colony formation assay, SW620 and SW480 cells (1 000 per well) were seeded into six-well plates. The culture medium in the culture plate was changed regularly, and cells were incubated for 15 days. After incubation, the six-well plates were washed with pre-cooled PBS and fixed with 4% paraformaldehyde. The cells were stained with 0.25% crystal violet (cat. no. C0121; Solarbio). Images of the stained colonies were captured after washing and natural drying, and the number of colonies was calculated using ImageJ software.

5-Ethynyl-2′-deoxyuridine (EdU) assay

For EdU incorporation assays, cells were seeded into 96-well culture plates until they reached 50–60% confluence on the next day. The proliferative capacity of COAD cells was assessed using the Cell-LightTM EdU Apollo567 In Vitro Kit (cat. no. C10310-1; RiboBio, China), following the manufacturer's instructions. Images of the stained cells were collected using a fluorescence microscope and analyzed using ImageJ software.

Transmission Electron Microscopy

SW620 cells were seeded into 10-cm plates and treated with or without RSL3 (5 μM) for 12 h. After treatment, the cells were trypsinized, collected, and fixed with 2.5% glutaraldehyde at room temperature in the dark for 2 h. A secondary fixation

was performed using 1% OsO4. Sheets (60–80 nm) were prepared after dehydration and stained with uranyl acetate and plumbous nitrate. Images were acquired using a transmission electron microscope (JEM1400PLUS; JEOL, Japan).

Evaluation of malondialdehyde (MDA) and glutathione (GSH) level

Cells were inoculated into 10-cm dishes and treated with specific concentrations of RSL3 for the indicated time periods. MDA levels were measured using the Lipid Peroxidation MDA Assay Kit, according to the manufacturer's instructions (cat. no. S0131S; Beyotime). The cells were inoculated into 10-cm dishes. GSH and oxidized glutathione (GSSG) levels were measured with the GSH and GSSG Assay Kit, following the provided protocol (cat. no. S0053; Beyotime).

Relative cystine uptake assay

For the cystine uptake assay, 1.5×10^5 cells were seeded into a black 96-well cell culture plate and incubated at 37 °C for 24 h in a 5% CO_2 incubator. Cystine uptake was determined according to the manufacturer's protocol (cat. no. UP05; DOJINDO, Japan).

FerroOrange staining assay

For the FerroOrange staining assay, 2×10^4 cells were seeded into 35 mm confocal dishes. Next, a diluted FerroOrange solution (1 μM) was added, and the cells were incubated for 25 min (cat. no. F374; DOJINDO). FerroOrange signals were detected using a fluorescence microscope. Images of the stained cells were collected and analyzed using ImageJ software.

Supplementary Table 1. The nucleotide sequences of the shRNAs .

	Nucleotide sequence (5′– 3′)
**Sh-HnRNPU#1**	CAGTGCTTCTTCCCTTACAAT
**Sh-HnRNPU#2**	GCAACTGTGAGACTGAAGATT
**Sh-HnRNPU#3**	AGGGAACTACAACCAGAACTT

Supplementary Table 2. The List of antibodies employed in this study.

protein	Manufacturer	Catlog No.	Application	Dilution
β-actin	ProteinTech	66009-1-Ig	Western blot	1:3000
GAPDH	ProteinTech	10494-1-AP	Western blot	1:3000
HSP90	ProteinTech	13171-1-AP	Western blot	1:2000
SLC3A2	Cell Signaling Technology	#47213	Western blot	1:1000
SLC7A11	ab307601	abcam	Western blot	1:1000
GPX4	ProteinTech	30388-1-AP	Western blot	1:1000
HNE	abcam	ab48506	Western blot	1:1000
FTH1	ProteinTech	11682-1-AP	Western blot	1:2000
TFRC	Cell Signaling Technology	#13113	Western blot	1:1000
Cyclin E1	ProteinTech	11554-1-AP	Western blot	1:1000
CDK2	ProteinTech	10122-1-AP	Western blot	1:3000
HnRNPU	ProteinTech	14599-1-AP	Western blot	1:2000
SLC3A2	Cell Signaling Technology	#47213	IF	1:300
SLC7A11	abcam	ab307601	IF	1:400
Ki-67	abcam	ab15580	IHC	1:200
4-HNE	abcam	ab48506	IHC	1:200
HnRNPU	ProteinTech	14599-1-AP	IHC	1:200

SLC3A2	Cell Signaling Technology	#47213	IHC	1:400
SLC7A11	abcam	ab307601	IHC	1:200

Supplementary Table 3. Cox-regression analyses.

Factors	B	SE	Wald	HR	95%CI		p value
					Lower	Upper	
**Univariate**							
Age	0.0003	0.0005	0.363	0.9997	0.998	1.000	0.547
Tumor size	0.385	0.399	0.933	0.680	0.311	1.486	0.334
LN metastasis	1.182	0.370	10.211	3.262	1.580	6. 737	0.001**
AJCC Clinical stage	1.361	0.375	13.163	3.899	1.869	8.131	0.0002**
Histological Grade	1.117	0.416	7.217	3.057	1.353	6.907	0.007**
TNM stage	0.647	0.732	0.781	1.910	0.455	8.022	0.377
HnRNPU	0.846	0.367	5.314	2.331	1.135	4.786	0.02*
**Multivariate**							
HnRNPU	1. 701	0.423	7.657	3.221	1.407	7.377	0.006**
Histological Grade	1.033	0.505	4.184	2.808	1.044	7.554	0.041*

* $p<0.05$, and ** $p<0.01$. *LN metastasis* lymph node metastasis. *B* Coefficient. *SE* standard error. *Wald* Wald statistic. *HR* hazard ratio. *CI* confidence interval.

Supplementary Figure & Figure legends

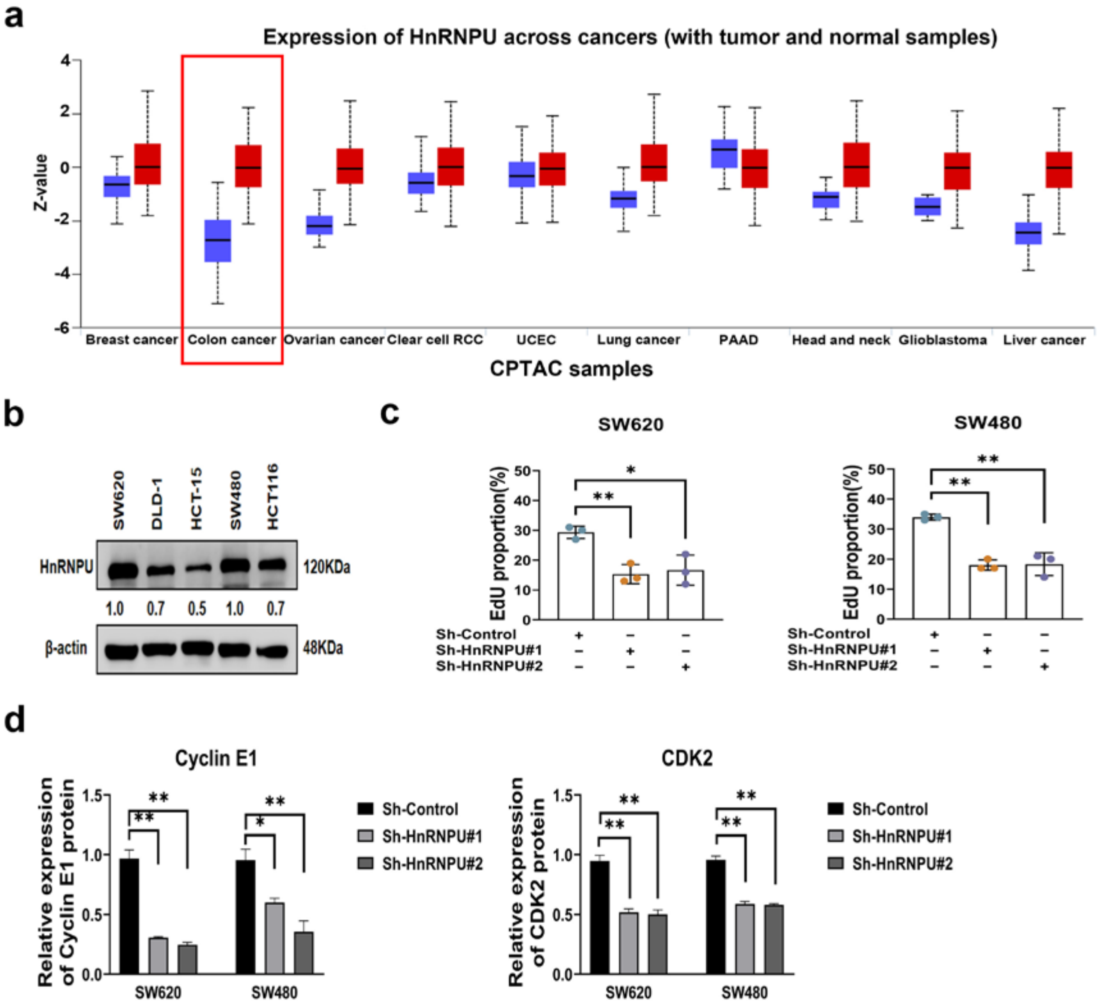

Supplementary Fig. 1 HnRNPU protein expression and quantitative results. (a) HnRNPU protein expression in normal and cancer tissues from the CPTAC database. **(b)** Western blot analysis of HnRNPU expression in SW620, HCT-15, SW480, DLD-1, and HCT116 cells. **(c)** Representative results of 5-ethynyl-2′-deoxyuridine staining. **(d)** Relative expression of Cyclin E1 and CDK2 was measured using Western blot analysis in COAD cells, after transfected with Sh-control, Sh-HnRNPU#1, and Sh-HnRNPU#2. *p*-value significant codes: *, $p < 0.05$; **, $p < 0.01$.

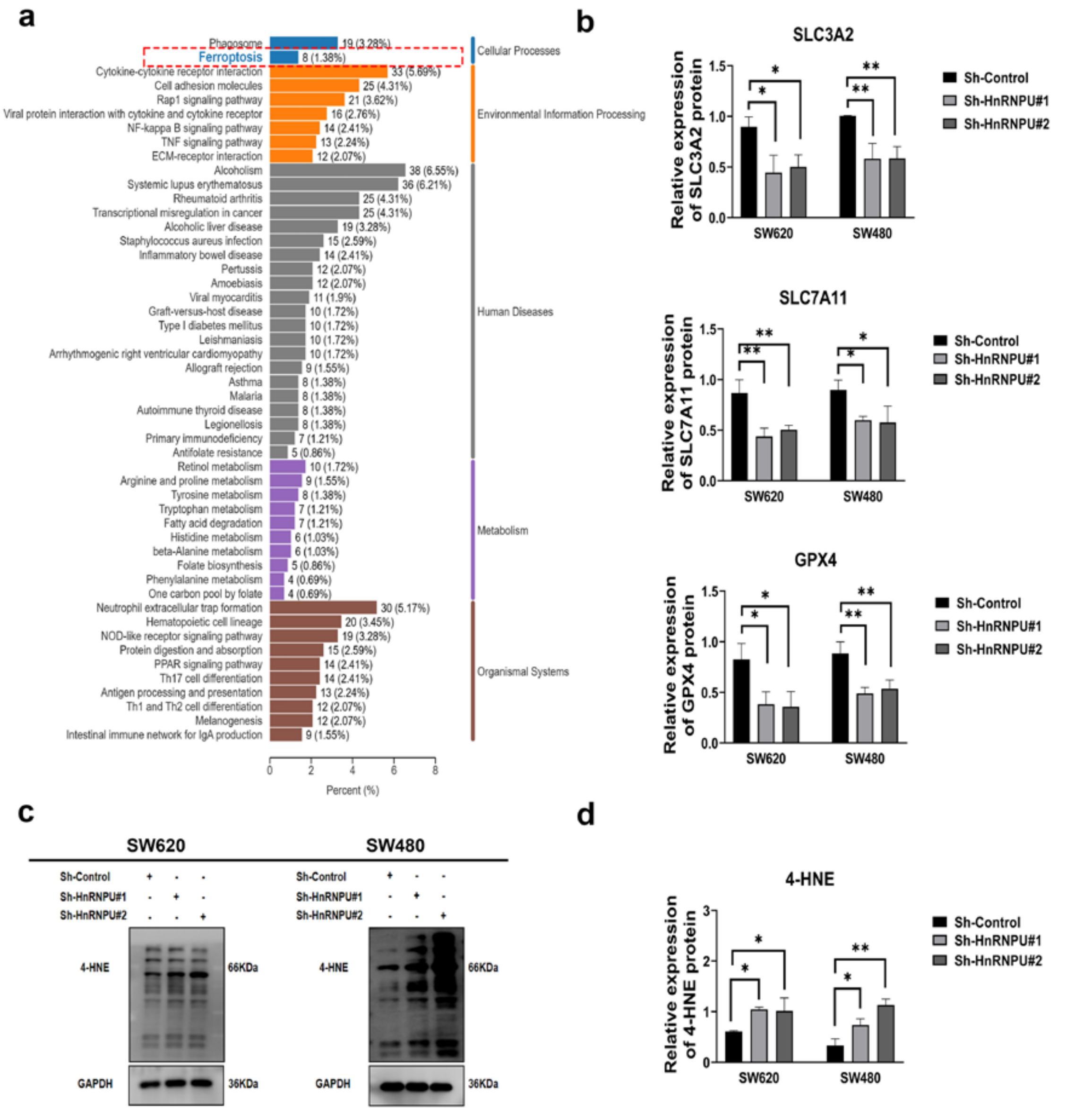

Supplementary Fig. 2 Pathway enrichment and Western blot analysis of SLC3A2, SLC7A11, GPX4, and 4-HNE expression in COAD cells. (a) Pathway enrichment analysis. **(b, c, d)** Relative expression of SLC3A2, SLC7A11, GPX4, and 4-HNE was measured using Western blot analysis in COAD cells, after transfected with Sh-control, Sh-HnRNPU#1, and Sh-HnRNPU#2. GAPDH was included as an internal control. *p*-value significant codes: *, $p < 0.05$; **, $p < 0.01$.

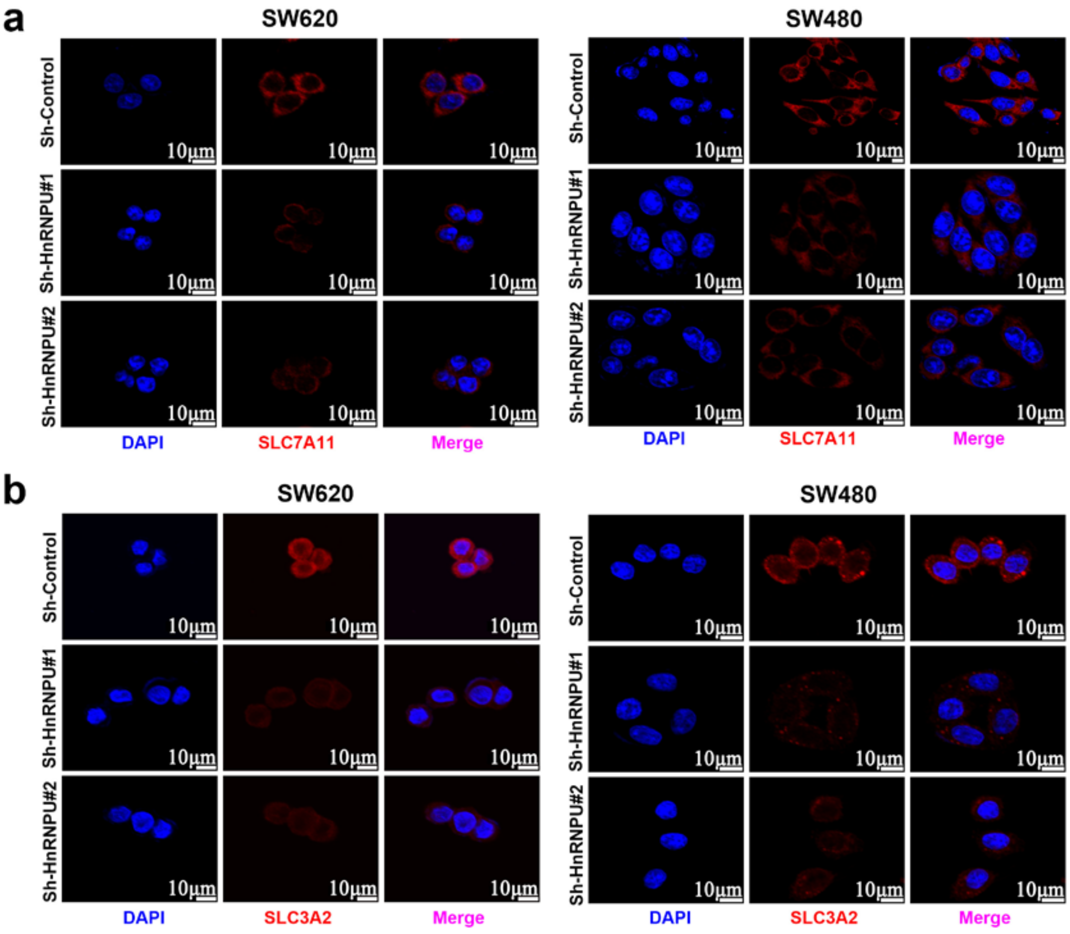

Supplementary Fig. 3 Immunofluorescence staining of SLC7A11/SLC3A2. (a, b) Immunofluorescence staining of SLC7A11 and SLC3A2 in HnRNPU-knockdown cells. Scale bar = 10 μm.

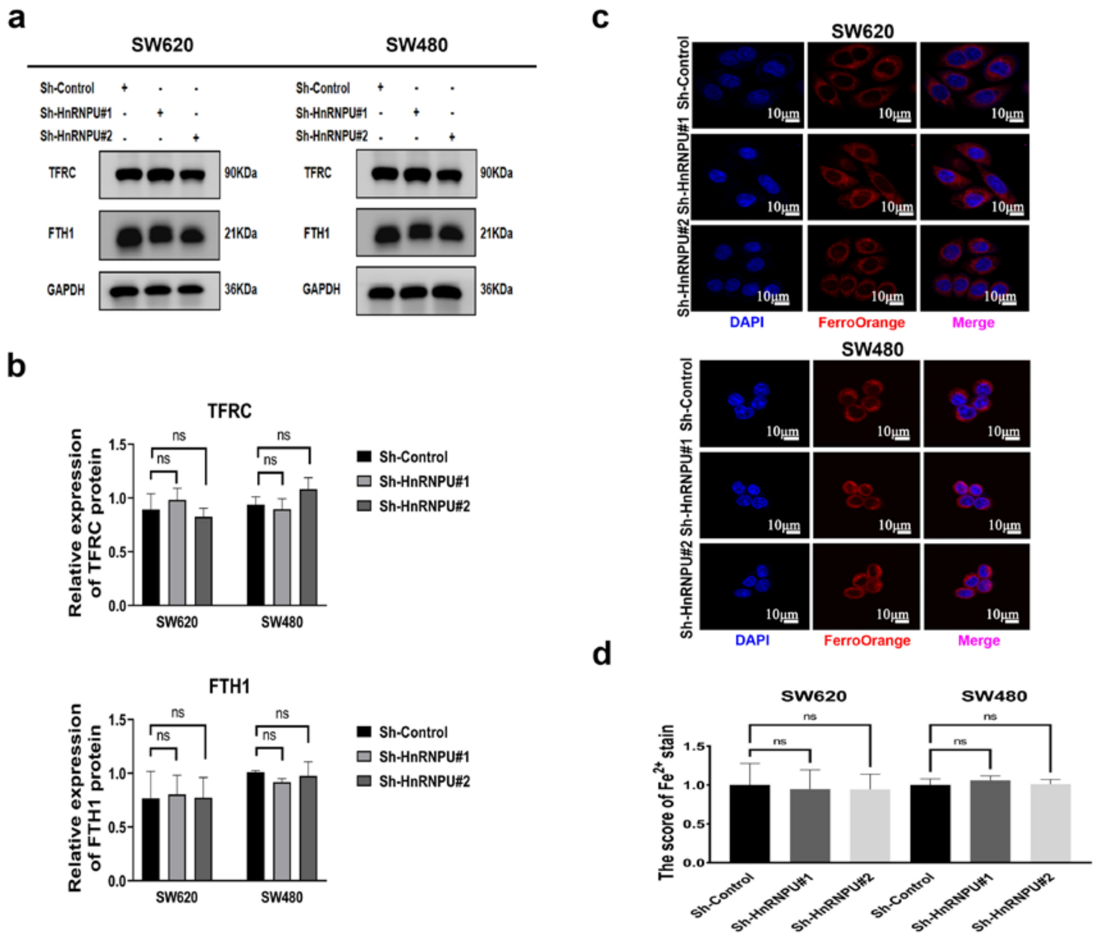

Supplementary Fig. 4 Western blot analysis of FTH/TFRC expression and FerroOrange staining. (a, b) Relative expression of FTH and TFRC was measured using Western blot analysis in COAD cells, after transfected with Sh-control, Sh-HnRNPU#1, and Sh-HnRNPU#2. GAPDH was included as an internal control. **(c, d)** FerroOrange staining was used to detect intracellular ferrous iron. Scale bar = 10 μm. *p*-value significant codes: ns, $p \geq 0.05$.

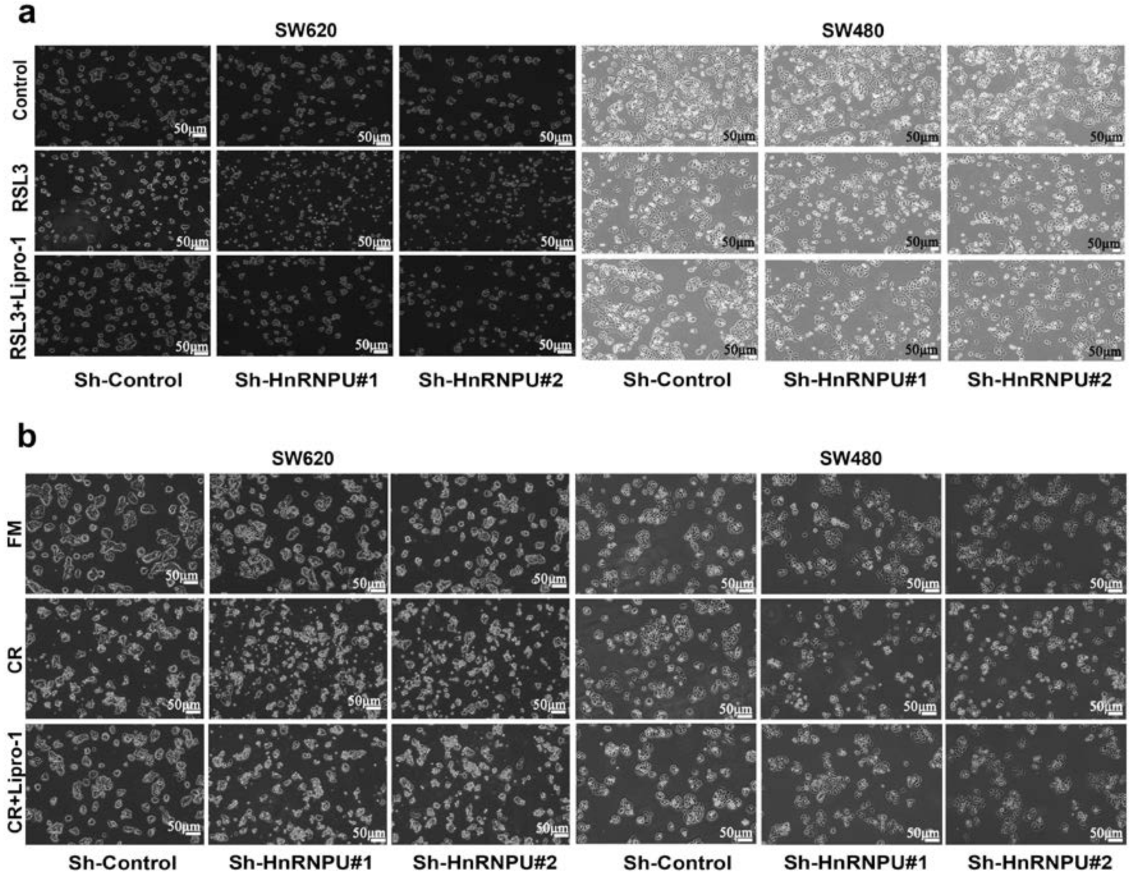

Supplementary Fig. 5 Micrographs of HnRNPU knockdown cells under RSL3/cystine restriction treatments alone or with liproxstatin-1. (a) Micrographs of HnRNPU knockdown cells with RSL3 alone or in combination with 1μM liproxstatin-1 treatment for 24 h. Scale bar = 50 μm. **(b)** Micrographs of HnRNPU knockdown cells cultured under cystine restriction alone or in combination with 1μM liproxstatin-1 treatment for 24 h. Scale bar = 50 μm.

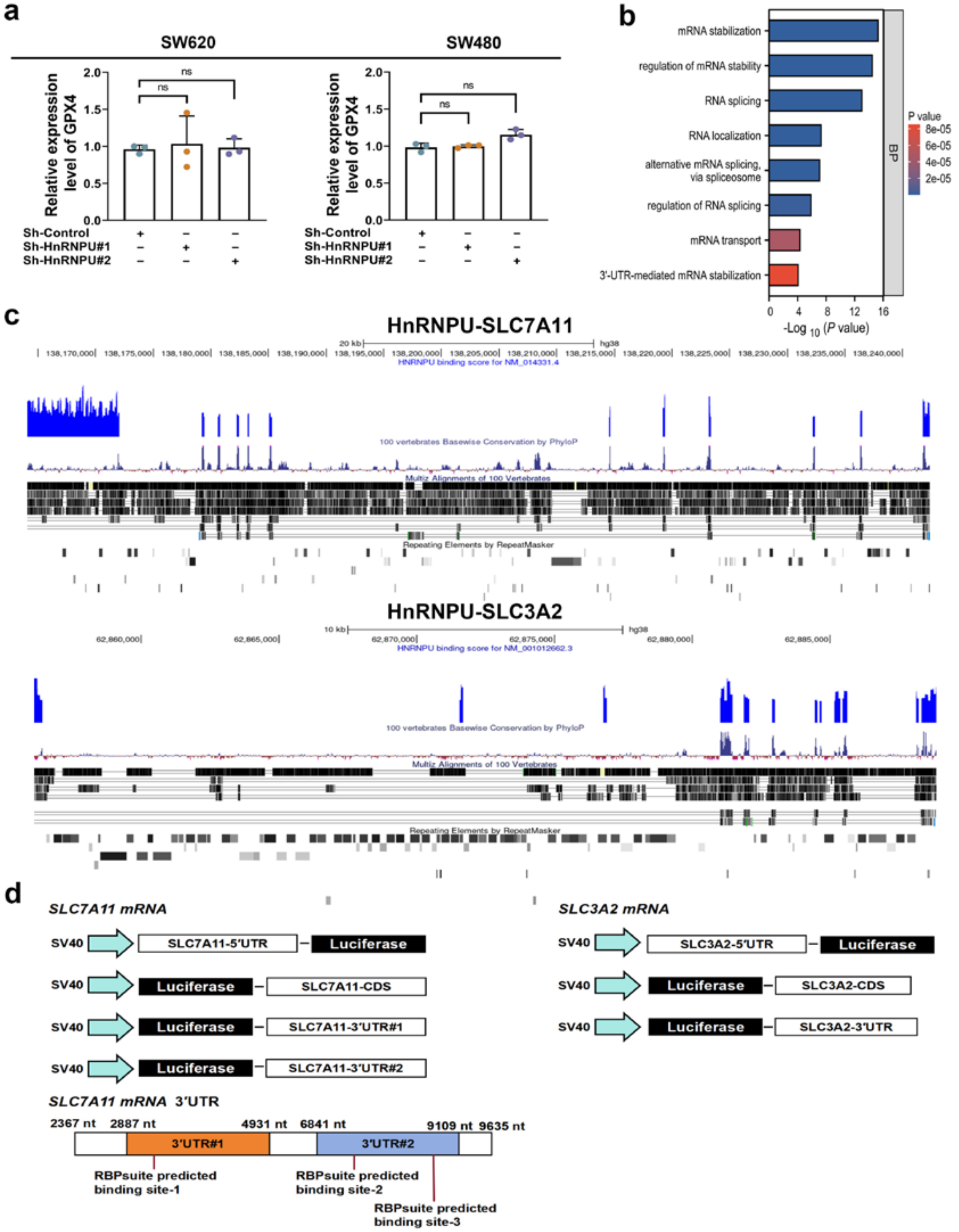

Supplementary Fig. 6 RT-qPCR analysis of GPX4 expression and database predictions for HnRNPU molecular function and binding sites with system xc$^-$. (a) RT-qPCR showing GPX4 expression in HnRNPU knockdown cells. **(b)** The STRING database was used to predict the molecular function of HnRNPU. **(c)** The RBPsuite database was explored to predict the binding sites between HnRNPU and system xc$^-$ (SLC3A2/SLC7A11). **(d)** Schematic presentation shows

three potential binding sites of HnRNPU in the SLC7A11-3′ UTR region predicted via the RBPsuite website. *p*-value significant codes: ns, $p \geq 0.05$.

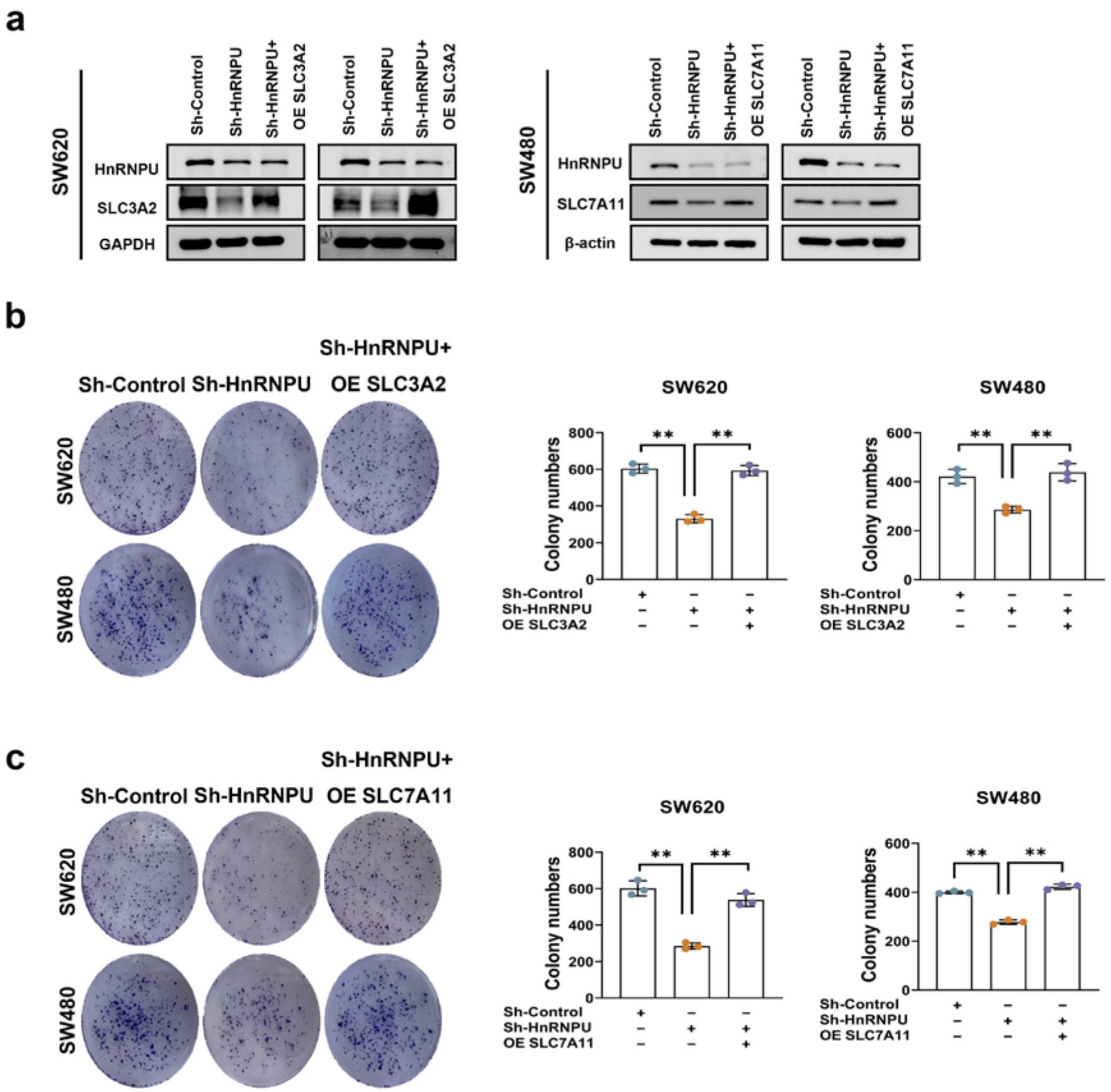

Supplementary Fig. 7 SLC3A2/SLC7A11 lentivirus efficacy, and colony formation in HnRNPU-knockdown cells. (a) Western blot analysis was used to confirm the transfection efficacy of SLC3A2 and SLC7A11 overexpression lentivirus in HnRNPU knockdown cells. GAPDH and β-actin was included as an internal control. **(b, c)** Colony formation assay was performed in HnRNPU knockdown cells with or without SLC3A2 or SLC7A11 overexpression. *p*-value significant codes: **, $p < 0.01$.
